# Perceptions, knowledge and adoption of artificial intelligence in rheumatology: results from a British Society for Rheumatology survey

**DOI:** 10.1093/rheumatology/keag454

**Published:** 2026-08-20

**Authors:** Hansel Canagarajah, Pratyasha Saha, Jordan Tsigarides, Nicholas Fuggle, Meghna Jani

**Affiliations:** Institute of Inflammation and Aging, University of Birmingham, Birmingham, UK; Norwich Medical School, University of East Anglia, Norwich, UK; Norwich Medical School, University of East Anglia, Norwich, UK; MRC Lifecourse Epidemiology Centre, University of Southampton, Southampton, UK; Centre for Epidemiology Versus Arthritis, Centre for Musculoskeletal Research, The University of Manchester, Manchester, UK; NIHR Manchester Biomedical Research Centre, Manchester University NHS Foundation Trust, Manchester Academic Health Science Centre, Manchester, UK; Department of Rheumatology, Salford Royal Hospital, Northern Care Alliance, Salford, UK

**Keywords:** artificial intelligence, machine learning, ambient scribes, digital readiness, data privacy

## Abstract

**Objectives:**

Artificial intelligence (AI) and machine learning applications are rapidly expanding across healthcare. Successful implementation of AI technologies in rheumatology will depend not only on technical performance but also on the perceptions and preparedness of end-users. This study evaluated the current opinions, expectations and concerns about AI among healthcare professionals and researchers in rheumatology across the UK.

**Methods:**

A 19-item survey was designed and distributed through national and regional networks aimed at the rheumatology workforce between June 2025 and January 2026, targeted at consultant rheumatologists, doctors-in-training, allied health professionals, specialist nurses and non-clinical researchers in rheumatology. The questions included respondent background data, current applications of AI in clinical care and research, opinions about AI in terms of perceived impact, concerns, educational needs and expected performance.

**Results:**

Of the 218 respondents, 39 to40% reported daily or weekly use of AI in research and clinical practice respectively. The most common clinical uses were using large language models (LLMs) to look up medical facts (45%), to improve grammar/spelling of clinical documentation (28%), to generate differential diagnoses (22%) and ambient scribe use (17%). Eighty-six percent anticipated that AI would substantially impact clinical practice in 5 years or less. Administrative tasks (85%) and musculoskeletal imaging (63%) were perceived as the areas likely to experience the greatest impact from AI. Highlighted concerns included data security/privacy (70%) and medical liability (70%), followed by lack of explainability (47%). One in four reported excellent confidence in using digital technology, with only 6% self-rating their AI knowledge as excellent. A strong interest in education about AI was expressed regarding several areas including the ethical and safe use of AI (66%), safe and efficient use of LLMs in clinical practice (64%), and use of ambient AI scribes (59%).

**Conclusion:**

AI is already being used frequently in UK rheumatology practice and research, with most anticipating a considerable impact on clinical care within the next 5 years or less. However, despite enthusiasm for adoption, important concerns regarding data security, liability and explainability remain, alongside low self-reported AI knowledge, highlighting the need for targeted education, robust governance and safe clinical implementation strategies.

*Rheumatology* key messages

- Up to 40% of respondents use artificial intelligence (AI) daily or weekly for clinical purposes including looking up medical facts, generating differential diagnoses and ambient scribe use.
- Participants reported high digital literacy, yet only a minority (6%) reported excellent AI-specific knowledge.
- Administrative support and musculoskeletal imaging were perceived areas of high AI-impact, with concerns including data security, medical liability and explainability.

## Introduction

Whilst artificial intelligence (AI) is often heralded as a revolutionary force in modern medicine, its integration into UK rheumatology departments remains largely aspirational, with its clinical potential yet to be realized. Although AI in rheumatology has demonstrated the capacity in research settings to enhance disease classification, image interpretation and clinical risk prediction, and to extract important insights from electronic health records through natural language processing [1–5], these applications have yet to transition into routine clinical practice. Beyond diagnostic and clinical decision support, generative AI systems such as ambient scribes that convert speech into structured medical documentation offer opportunities to automate administrative tasks including clinic letter generation [6]. This prospective evolution aligns with a policy environment increasingly focused on digitally enabled service transformation, as evidenced by the NHS 10-Year Health Plan’s ambition to shift from analogue to digital [7]. However, for rheumatology to successfully bridge the gap between innovation and implementation, the preparedness of the speciality’s workforce is of the utmost importance.

The successful adoption of these technologies within the UK is contingent upon factors far exceeding simple algorithmic performance. As AI systems move from administrative assistance toward influencing clinical judgement, the challenge becomes whether they can be introduced in a way that is safe, transparent and trustworthy. This is particularly relevant within the National Health Service (NHS) framework, where considerable uncertainty remains regarding professional accountability when AI-supported decisions contribute to adverse outcomes [8, 9]. Recent national guidance on ambient scribes underscores this risk, illustrating that even seemingly low-risk tools can introduce clinically significant errors if oversight and data governance are inadequate [6]. Consequently, the perceptions and preparedness of the rheumatology workforce are not merely a matter of opinion but are the primary gatekeeper for adoption and responsible implementation of any future technology.

Previous UK survey data also suggest that AI use is no longer hypothetical: increasingly doctors across specialities are using AI, particularly general practitioners, report growing use of generative AI for documentation and clinical reasoning support [10, 11]. However, specialty-specific evidence in rheumatology remains comparatively limited, particularly from the UK. A recently published EMEUNET (The Emerging EULAR Network) international survey amongst rheumatology professionals showed considerable differences in uptake of AI across continents and countries, with a minority of responses from the UK [12], demonstrating an unmet need to characterize UK-specific practice especially given the differences in digital landscape and regulations. Current evidence suggests that attitudes and reported use of AI may be evolving rapidly, but may also differ according to geography, professional mix and survey timing. Important questions therefore remain about the current UK rheumatology landscape including, (i) how is AI currently being used in clinical practice and research, (ii) which applications are seen as likely to have the greatest impact, (iii) what concerns dominate, (iv) what level of performance clinicians would consider acceptable for predictive systems and (v) whether there is a clear need for formal education on the ethical, safe and secure use of AI.

This study therefore aims to evaluate the current landscape of perceptions, knowledge and adoption across rheumatology. By identifying the current views of the rheumatology workforce, specific drivers and barriers within this diverse population, we provide a foundation for a roadmap that aligns technological potential with the practical, clinical realities of UK rheumatology. Although focused on the UK healthcare system, these findings provide an international case study for anticipating and managing similar technological transitions elsewhere.

## Methods

### Study design

A cross-sectional survey was designed to evaluate the perceptions and current usage of AI within the rheumatology community. The 19-item questionnaire was developed through a collaborative input from consultant rheumatologists, clinical researchers, allied health professionals and specialist nursing staff to ensure the survey was clinically relevant and comprehensive. Survey questions were uploaded to the Qualtrics platform from The University of Manchester, with results collected electronically. Ethics approval was not required as no personal identifiable information, sensitive information or data from vulnerable groups was collected.

### Respondent recruitment and distribution

The survey was disseminated to a broad cohort of UK-based rheumatology healthcare professionals and researchers through established national and regional networks between 30 June 2025 and 31 January 2026. Targeted respondents included consultants, specialist trainees, specialist nurses, pharmacists and non-clinical researchers within the field of musculoskeletal (MSK) medicine.

### Survey domains

The survey was structured into three primary domains: (i) respondent background and demographic data, (ii) current clinical and research applications of AI and machine learning and (iii) professional opinions on AI. Demographic variables included professional role, gender identity, age band and years of specialist experience. To assess digital literacy, respondents self-rated their confidence in utilizing digital health technology and their relative knowledge of AI applications compared with their peers. The survey utilized diverse response formats, including multiple choice, ranking of statements and free-text questions. Specific clinical applications were explored, including use of large language models (LLMs) (e.g. ChatGPT, Gemini, Claude) and ambient scribing tools (e.g. TORTUS, Heidi). Additionally, the survey captured perceptions of acceptable error thresholds for AI-based treatment outcome predictions and ranked respondent concerns across domains such as data security, medical liability and the ‘black box’ nature of diagnostic outputs.

### Data analysis

Quantitative data were analysed using descriptive statistics to summarize respondent characteristics and response distributions. The survey included questions relevant to different professional roles (e.g. clinicians, researchers), and therefore some items may not have been applicable to all respondents. Partial responses were retained for analysis to maximize response rates and reduce non-response bias. To reflect this, we report item-level completion rates with denominators presented with proportions.

Statement ranking questions were further stratified to aid visualization of the data. Frequency of each rank position was subcategorized into three ordinal priority categories: high priority (ranked 1st–3rd), medium priority (ranked 4th–6th) and low priority (ranked 7th–9th). For each priority category, frequencies of high, medium and low were expressed as a percentage of total respondents. A radar plot of high, medium and low percentages was plotted to highlight key areas. In addition, median rank was used as a measure of central tendency and to enable comparison of relative importance across variables. Qualitative data from free-text comments were analysed using a reflexive thematic analysis approach [13], to provide further context. R Studio Version 2025.09.2 + 418 was used to generate visualizations.

## Results

A total of 218 respondents took part in the survey, with baseline characteristics summarized in Table 1. The majority were female (58%, *n* = 127/218), and two-thirds were aged between 36 and 55 years (67%, *n* = 147/218).

**Table 1:** Baseline characteristics of respondents.

Characteristics	Respondents (*n* = 218), *n* (%)
Gender	
Male	89 (41)
Female	127 (58)
Prefer not to say	2 (1)
Age (years)	
≤35	43 (20)
36–45	73 (33)
46–55	74 (34)
56–65	24 (11)
66–75	4 (2)
Profession	
Consultant in rheumatology	106 (49)
Specialist trainee in rheumatology	32 (15)
Specialist nurse	18 (8)
Pharmacist	5 (2)
Allied health professional	17 (8)
GP with an interest in treating MSK conditions	1 (0)
Non-clinical researchers within rheumatology/musculoskeletal medicine	17 (8)
Other	22 (10)
Subspecialty	
Adult rheumatology	190 (87)
Child and adolescent rheumatology	4 (2)
Both	6 (3)
Years of practice/experience in rheumatology	
Currently in speciality training	28 (13)
PhD student	5 (2)
<5 years	32 (15)
5–10 years	35 (16)
11–20 years	56 (26)
21–30 years	34 (16)
>30 years	12 (6)

GP: general practitioner; MSK: musculoskeletal.

### Current applications of AI in clinical practice

**Figure 1:**
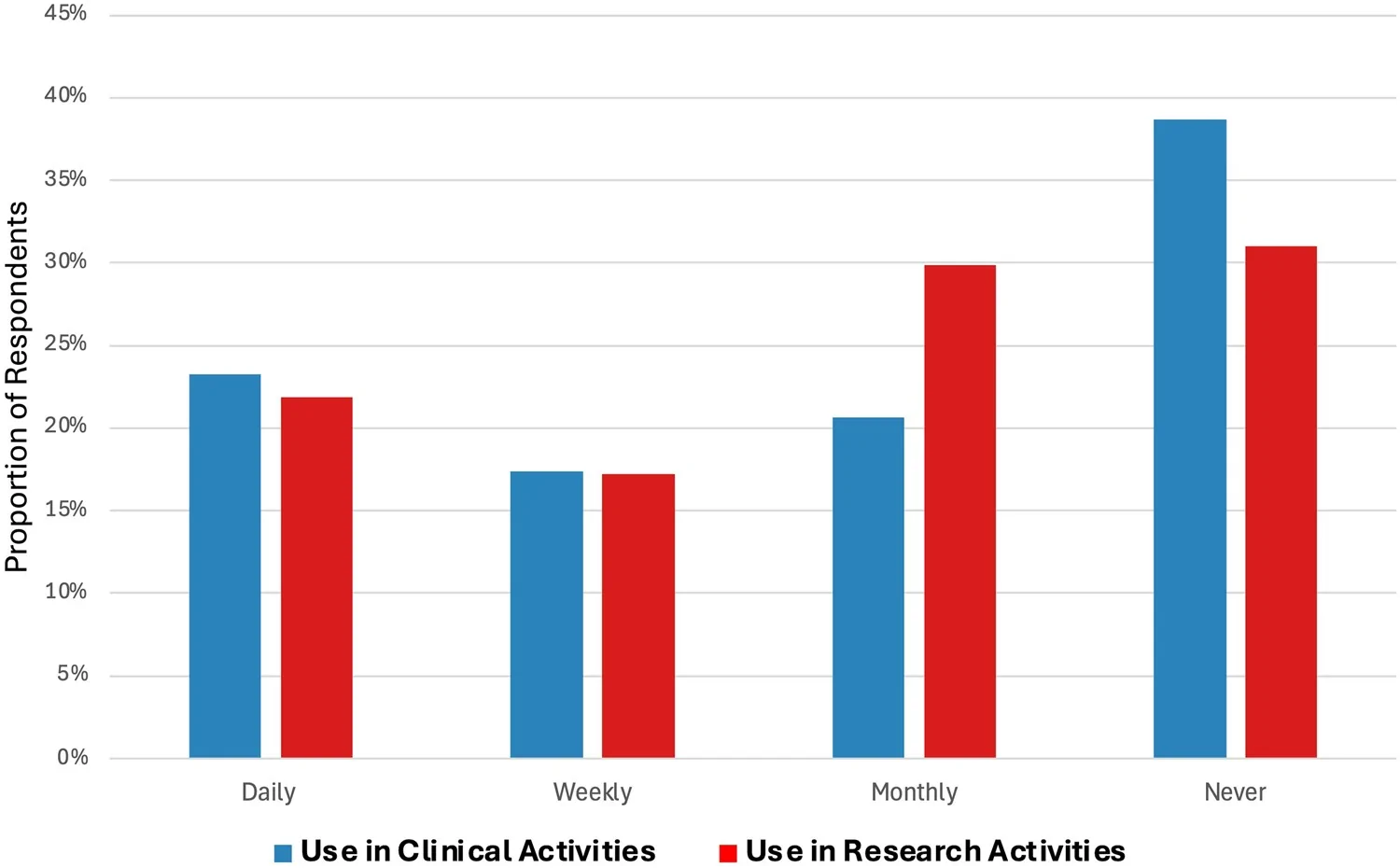
Frequency of artificial intelligence in clinical practice and research

Use of AI in clinical practice was common, although frequency varied in clinical and research settings (Fig. 1). In clinical practice approximately one in four respondents (23%, *n* = 36/155) reported daily use, while 17% (*n* = 27/155) reported weekly use, and 21% (*n* = 32/155) monthly use. Over a third (39%, *n* = 60/155) of respondents reported never using AI clinically. Among research-active respondents, AI use in research was also variable: 22% (*n* = 19/87) using AI daily, 18% (*n* = 15/87) weekly, 30% (*n* = 26/87) monthly, and 31% (*n* = 27/87) never having used AI for research.

LLMs were the most frequently used AI tools (66%, *n* = 114/173). ChatGPT was used by 45% (*n* = 78/173), followed by Gemini (10%, *n* = 17/173), Claude (3%, *n* = 5/173) or other LLMs (8%, *n* = 14/173). Ambient scribe use was also reported in 17% (*n* = 30/173); most commonly Heidi (14%, *n* = 25/173), with limited use of TORTUS (1%, *n* = 1/173) or other ambient tools (2%, *n* = 4/173). Few respondents reported using AI-based clinical decision support systems (2%, *n* = 4/173) or AI-based diagnostic tools (2%, *n* = 3/173). Approximately one in four respondents (23%, *n* = 40/173) reported using AI to assist with teaching. Over a third of respondents (38%, *n* = 65/173) reported not using any AI tools clinically.

AI was most frequently used to look up medical facts (45%, *n* = 78/173) and to improve grammar, spelling or syntax of clinical documentation (28%, *n* = 49/173), and 21% (*n* = 36/173) reported using AI to generate differential diagnoses in complex cases. AI was less commonly used to generate treatment plans (8%, *n* = 13/173). Ambient recording tools in general (including for meeting documentation as well as letters) was reported by 27% (*n* = 46/173). Other AI tools used for clinical purposes included Microsoft Copilot (4%, *n* = 7/173), OpenEvidence (1%, 2/173), DeepSeek, Whisper Transcript AI and ChatGPT (*n* = 1 each). Few respondents were currently using AI-based clinical decision support systems (2%, *n* = 4/173) and were using AI-based diagnostic tools such as for imaging analysis or early detection of diagnosis (2%, *n* = 3/173), which included Esaote (e-SPADES), an AI-powered imaging software. A small proportion were already using AI tools that were already incorporated into the electronic health record (5%, 8/173). These companies included EPIC (*n* = 2), Microsoft Dragon integrated with EPIC (*n* = 1), Carebit (*n* = 1) and epro (*n* = 2).

In the free-text section, responses included using the ‘AI overview that is generated when you search Google’ and similar responses for checking clinical facts. Respondents reported a variety of additional clinical uses for AI tools, primarily related to communication and patient education, including ‘checking clinic letters are appropriate for educational level for the patient’. Further analysis of the free-text comments is provided in Supplementary Data S1.

### Current implementation in clinical practice

Fewer than one in five (18%, *n* = 28/159) respondents reported that AI tools had been implemented and were actively used in their clinical workplace. However, 21% (*n* = 33/159) of respondents reported that their organization was in an exploratory phase without having implemented AI tools in clinical practice yet, and a further 20% (*n* = 32/159) were aware of preliminary discussions in place. In contrast, 29% (*n* = 46/159) of respondents were not aware of any interest in AI implementation in their trust, and 13% (*n* = 20/159) reported that they did not know.

### Current applications of AI in research

In research settings, AI was most used to improve grammar and clarity of draft manuscripts (57%, *n* = 38/67) and to assist with literature searching (57%, *n* = 38/67). Additional uses included brainstorming new research ideas (48%, *n* = 32/67), assistance with research paper writing (37%, *n* = 25/67), data analysis (33%, *n* = 22/67) and writing statistical code (34%, *n* = 23/67). Assistance with grant applications was less frequently reported (18%, *n* = 12/67). Free-text responses highlighted that AI was also being used for research administrative tasks such as writing emails to collaborators and some self-education purposes such as summarizing research papers.

### Confidence in using digital technologies and AI in rheumatology practice

Self-reported confidence in using digital technology to access health information was high among respondents, with most reporting above-average confidence (42%, *n* = 92/218). One in four reported excellent confidence in using digital technology (23%, *n* = 51/218). In comparison, self-rated knowledge of AI and its application in clinical practice or research was lower overall. Relatively few described their knowledge in AI as excellent (6%, *n* = 10/175). Relative to their colleagues, most respondents rated their knowledge in AI as average (46%, *n* = 80/175).

**Figure 2:**
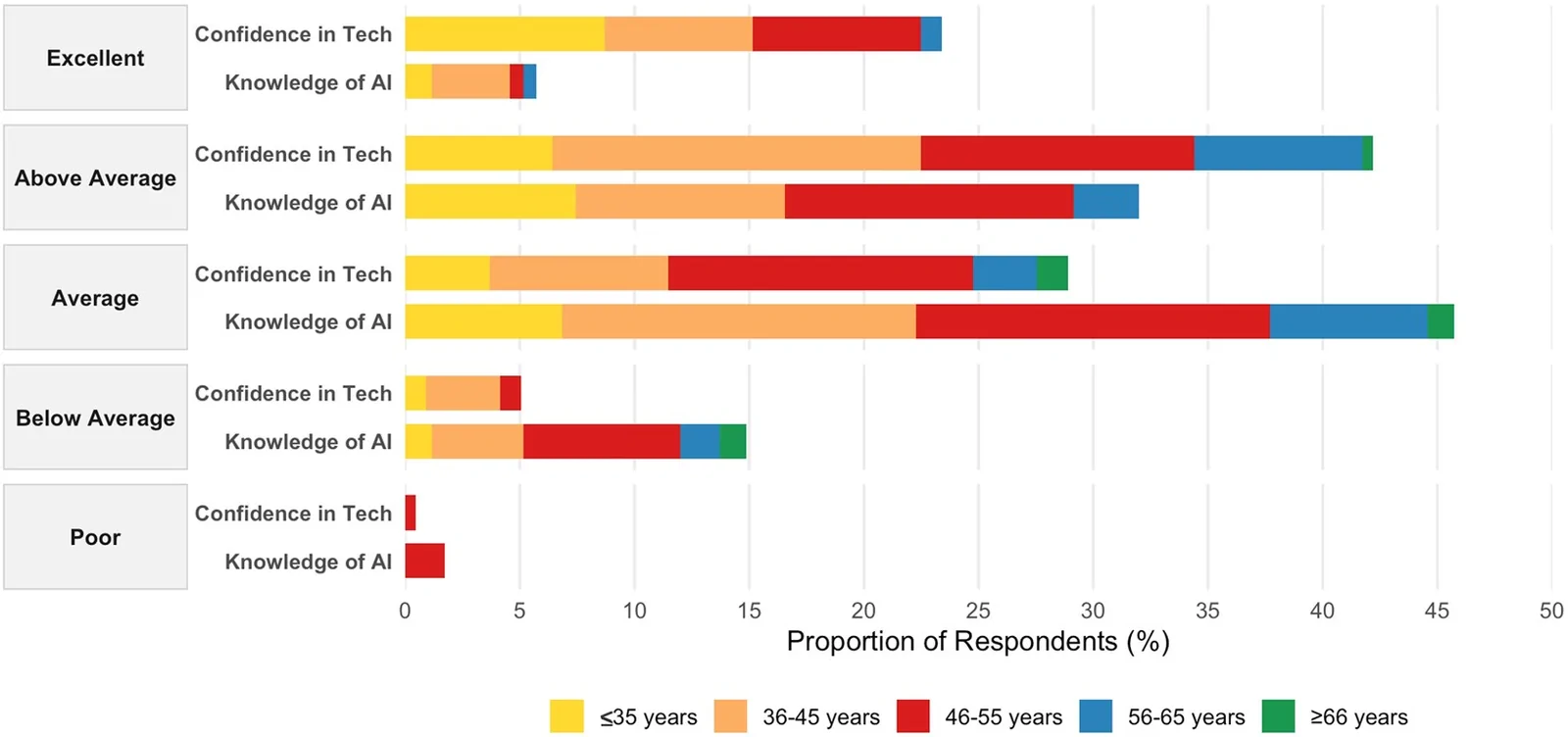
Self-reported confidence in using digital technology to access health information compared with self-reported knowledge of AI and its application in clinical practice stratified by age. Abbreviations: AI: Artificial Intelligence; Tech: Digital Technology

Age-stratified comparison of confidence in digital technology in comparison with knowledge of AI is summarized in Fig. 2. There was a trend towards higher confidence in using digital technology among younger respondents (≤35 years and 36–46 years age groups). Among those aged ≤35 years, the most frequent response was excellent confidence (44%, *n* = 19/43), and respondents aged 36–45 years commonly reported above-average confidence (48%, *n* = 35/73). In contrast, only two respondents aged ≥56 years reported excellent confidence (7%, *n* = 2/28).

When stratified by age, average knowledge of AI relative to colleagues remained the most common response across all age groups, aged ≤35 (*n* = 12/29, 41%), 36–45 (*n* = 27/56, 48%) and 46–55 (*n* = 27/65, 42%) years. Only a small number of those aged ≤35 years reported excellent knowledge of AI (*n* = 2/29, 7%).

### Anticipated timeline for AI use in rheumatology clinical practice

The majority (86%, *n* = 152/175) expected an impact within the next 5 years or sooner. Of these, 17% (*n* = 30/175) believed AI would have an immediate impact, 34% (*n* = 60/175) anticipated impact within 1–2 years and 35% (*n* = 62/175) expected impact within 5 years. A smaller number anticipated a slower impact of AI: within 10 years (7%, *n* = 12/175), beyond 10 years (1%, *n* = 2/175) or were not sure (5%, *n* = 8/175).

### Perceived areas of future impact of AI in rheumatology

**Figure 3:**
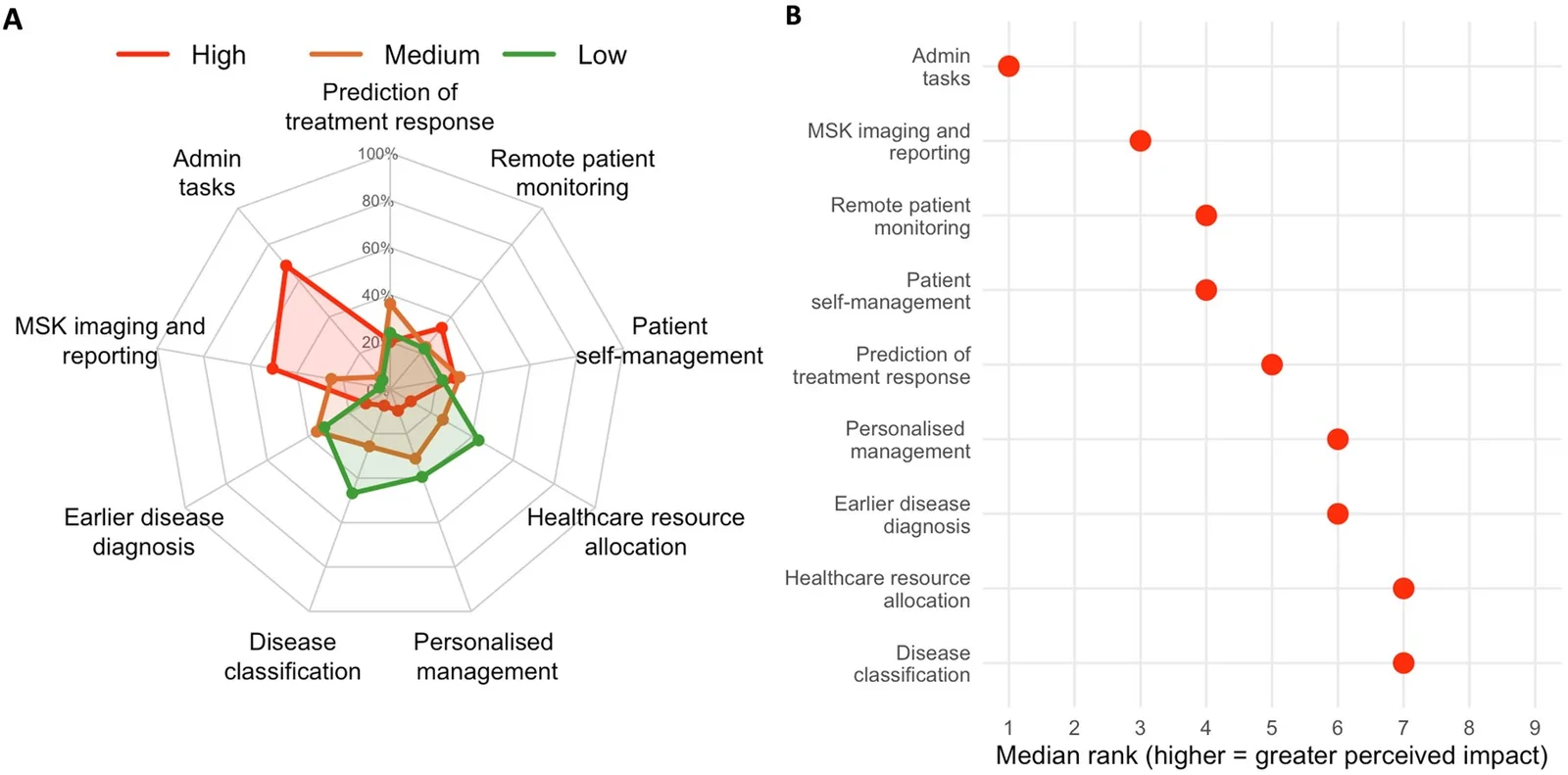
Perceived areas of future impact of artificial intelligence in rheumatology. (**A**) Perceived areas of future impact were ranked, high, medium and low priority. (**B**) Perceived areas of future impact were ranked using median rank. MSK: musculoskeletal

Administrative tasks were most frequently ranked as a high-priority area for AI impact, with 85% (*n* = 149/175) of respondents ranking this domain within the top three positions (Fig. 3). MSK imaging and radiology reporting was also frequently prioritized, with 63% (*n* = 110/175) ranking this as high priority. Prediction of treatment response (45%, *n* = 79/175), earlier disease diagnosis (45%, *n* = 78/175), personalized disease management (49%, *n* = 86/175) and remote patient monitoring (29%, *n* = 51/175) were considered medium impact areas, all of which all had a similar distribution of rankings (Fig. 3).

### Perceived concerns regarding the future use of AI in rheumatology

**Figure 4:**
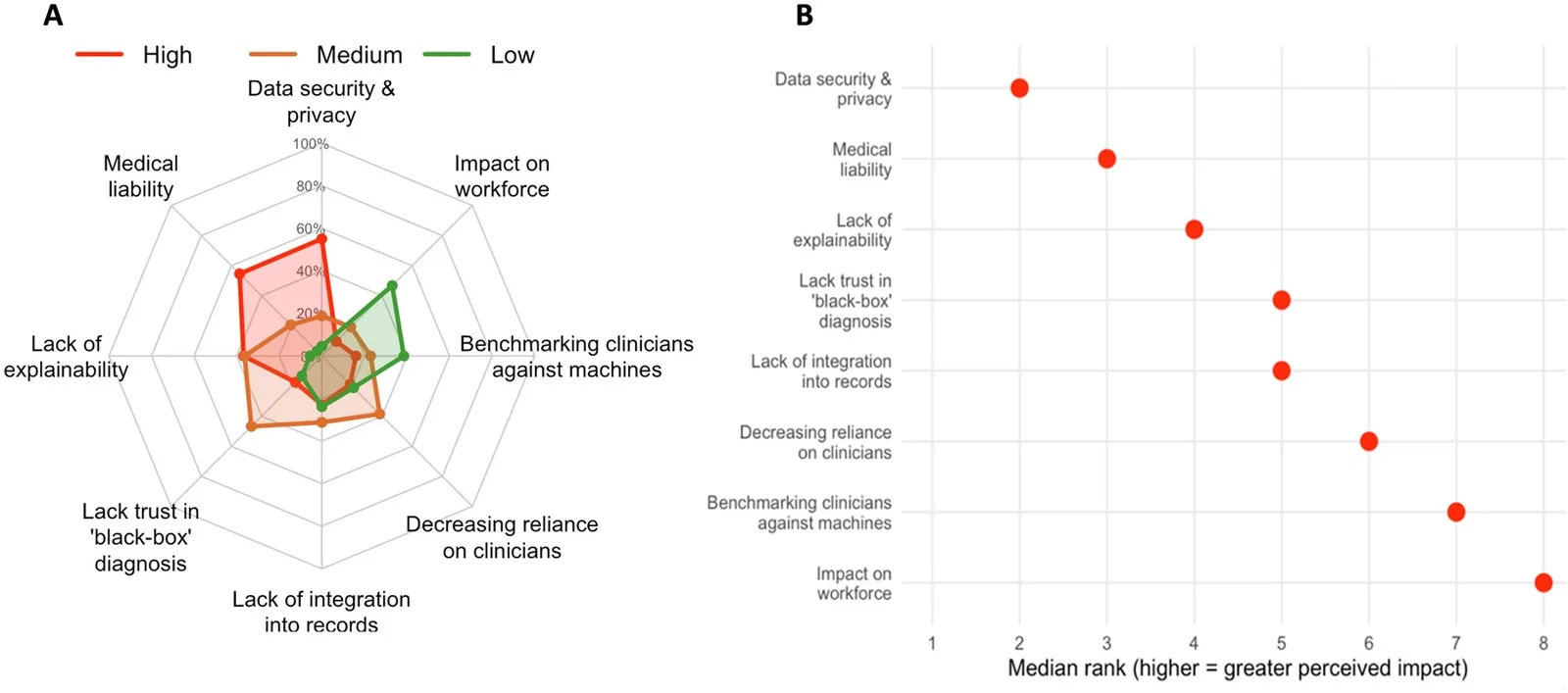
Perceived concerns regarding the use of artificial intelligence in rheumatology by median rank. (**A**) Perceived concerns regarding artificial intelligence were ranked, high, medium and low priority. (**B**) Perceived concerns regarding artificial intelligence using median rank

When asked about the concerns regarding the use of AI in rheumatology, data security and privacy (70%, *n* = 120/171), and medical liability (70%, *n* = 119/171) were the two domains most frequently ranked as high priority concerns (Fig. 4). Lack of explainability was split in rankings, with a high concern reported by 47% (*n* = 80/171) of respondents and as medium concern by 46% (*n* = 79/171). Lack of trust in ‘black box’ diagnosis was more frequently ranked as medium concern (60%, *n* = 102/171), alongside lack of integration into current records (40%, *n* = 68/171) and decreasing reliance on clinicians (49%, *n* = 84/171) (Fig. 4).

### Key areas for further education in AI

The most selected areas for further education in AI were ethical, safe and secure use of AI (66%, *n* = 116/175), safe and efficient use of LLMs in clinical practice (64%, *n* = 112/175), and use of ambient AI tools in clinical practice (59%, *n* = 104/175). Interest was also high in AI-based clinical decision support systems (58%, *n* = 101/175) and use of LLMs for research purposes (42%, *n* = 73/175). Fewer respondents selected computer vision and AI imaging applications (35%, *n* = 62/175), and only a minority indicated no interest in further AI education (5%, *n* = 8/175). Additional free-text analysis is provided in Supplementary Data S1.

### Acceptable performance thresholds for AI systems predicting treatment outcomes

Survey respondents were asked to select an acceptable performance threshold for AI systems used for predicting treatment outcomes as an example use case. Expectations were high, with 71% expecting AI to perform better than an average clinician. Specifically, one in five respondents (20%, *n* = 35/175) expected AI to outperform the best-performing clinician, and a further 22% (*n* = 38/175) expected AI to be equivalent to the best-performing clinician. Additionally, 29% (*n* = 51/175) expected AI to perform better than average performing clinician. In contrast, 26% (*n* = 46/175) considered equivalent performance to the average performing clinician acceptable and only a small minority (3%, *n* = 5/175) would accept performance at the level of the worst-performing clinician.

### Thematic analysis of free-text comments

Thematic analysis of free-text comments identified seven key themes, including safety, accuracy and trust concerns, application and benefits, and future direction and adoption, as shown in Table 2. Overall perceptions of AI reflected cautious optimism, with some opinions suggesting dissent (Table 2, Supplementary Data S1).

**Table 2:** Thematic analysis of free-text comments regarding the use of AI in rheumatology.

Theme	Description	Example quotes
1. Overall perceptions of AI	Broad attitudes toward AI, ranging from optimism to caution, with recognition of its growing role in healthcare	‘AI is a fantastic tool and should be embraced but with caution’ ‘Helpful, but uneasy in early stages’ ‘AI is here to stay’ ‘AI should be avoided at all costs’ ‘I feel overwhelmed by AI’
2. Safety, accuracy and trust concerns	Major concerns about hallucinations, reliability and potential risks to patient safety, alongside the need to preserve human clinical judgement	‘Hallucination is definitely real… content needs to be checked’ ‘No current way to safety net AI confabulation’ ‘The human factor is very important’
3. Governance, validation and regulation	Strong emphasis on the need for oversight, validated tools and clear governance frameworks before widespread adoption	‘Concerned about AI implemented without thorough testing’ ‘Would like approved NHS AI tools…’
4. Applications and benefits of AI	AI seen as valuable for reducing administrative burden, supporting clinical decisions, and improving patient engagement	‘Help with admin work… free us up for diagnostics’ ‘Support disease monitoring… detect early changes’ ‘Chat-based interactions for patients would be helpful’
5. Implementation challenges (workforce and systems)	Barriers to adoption including funding, infrastructure, integration issues, and limited digital literacy or training	‘Funding issues have impinged on this improvement’ ‘Integration is our next step’ ‘Digital literacy is low in the NHS’
6. Impact on clinical practice and patients	AI is changing clinician roles and patient expectations, but is largely viewed as a support tool rather than a replacement	‘ ‘‘ChatGPT told me…”—how do we manage this?’ ‘AI cannot replace clinical staff’
7. Future directions and adoption	Recognition of ongoing innovation and the need for clinician involvement in shaping future AI use	‘Currently leading an AI project…’ ‘Doctors should have a seat at the table’

AI: artificial intelligence; NHS: National Health Service.

## Discussion

This national survey provides valuable insight into the perceptions and knowledge of AI in rheumatology clinical practice and research in the UK. One in four respondents reported daily AI use in clinical practice, with LLMs being the most commonly used tools. Use of AI was diverse, from looking up medical facts using LLMs, using AI scribes and less frequently for imaging reports. Although confidence in digital technologies was high, AI-specific knowledge was lower, suggesting digital literacy has not translated into AI readiness. Most participants anticipated that AI will have a noticeable influence on rheumatology practice within the next 5 years, particularly in administrative processes and MSK imaging. A strong interest was expressed in education on the ethical, safe and secure use of AI, reflecting concerns regarding data governance, privacy and clinical accountability. Although many clinicians are already using AI tools in clinical or research activities at an individual level, implementation at an institutional level appears much more limited.

Our survey highlights the prevalent use of AI for several research and clinical tasks in the UK, with 39% and 40%, respectively, reporting daily or weekly use of at least one AI-enabled tool. This level of adoption appears higher than that reported in a previous national survey of German rheumatologists, in which 73% reported no prior work-related AI use [14], yet remains lower than the broader overall international uptake described in the EMEUNET survey, where 86.7% of respondents reported some use of AI in clinical practice or research [12]. Differences between studies likely reflect geography, survey timing and respondent characteristics. The German survey (2024) included board-certified rheumatologists and residents [14], whereas the EMEUNET survey (March–July 2025), disseminated via social media, likely recruited a younger, more digitally engaged population [12]. These differences limit direct comparisons, especially during a period of extravagant evolution of AI.

A noteworthy observation is the overwhelming preference for LLMs, specifically ChatGPT, which was utilized by 45% to look up medical facts. This reflects the ‘consumerization’ of medical AI, where general-purpose tools are repurposed for clinical documentation and medical fact-finding, by both patients as well as healthcare professionals [15]. This mirrors policy concerns highlighted by the National Institute for Health and Care Excellence (NICE) [16] and the Medicines and Healthcare products Regulatory Agency (MHRA) [17], which have called for clearer frameworks regarding the use of non-regulated software in high-stakes decision-making. Whilst 21% of our cohort used AI to generate differential diagnoses, the relatively low uptake for treatment plan generation (8%) perhaps suggests a healthy scepticism or a perceived limitation in the capability of current LLMs to handle complex longitudinal management for health purposes. These findings highlight the need for specialty-specific guidance on AI use.

The emergence of ambient scribes for transcription of clinic letters (17%), such as Heidi, indicates a burgeoning interest in potentially mitigating the administrative burden of clinical practice. This aligns with the NHS Long Term Plan’s emphasis on digital transformation to improve workforce retention and reduce burnout [7]. However, the disparity between the use of AI for administrative tasks versus its use in diagnostic tools or clinical decision support systems (2%) is stark. A notable finding from our survey is the mismatch between future expectations of AI performance in such a use case (prediction of treatment outcomes) and current levels of knowledge and use. Despite the majority (71%) expecting performance of AI to perform better than the average clinician, 39% of respondents have never used AI tools in clinical practice. Together, these findings suggest a substantial implementation gap between regulated AI devices and general-purpose LLMs, reflecting limitations in digital infrastructure, evaluation, funding and workforce education.

Our survey also highlights an important disconnect between confidence in using digital technology and knowledge of AI among rheumatology professionals. Whilst 65% of respondents in our survey reported above average or excellent confidence in digital technology, only 6% rated their AI knowledge as excellent. Knowledge of AI was also limited among younger respondents, highlighting the need for education across the workforce, including trainees. The need for workforce development in this field has been recognized in recent years. The 2019 Topol Review [18] that called for an NHS workforce that is both able and willing to use AI and related digital technologies. Subsequent work by NHS England [19] and the NHS AI Lab [20] further identified the need for better training on how to use AI as a core requirement for safe and effective adoption. Our findings support targeted AI education to bridge the gap between digital literacy and AI readiness, particularly regarding ethical, safe and effective use.

Despite the knowledge gap, there remains strong overall optimism for the near-term impact of AI, with 86% anticipating a meaningful effect on clinical practice within 5 years. In our cohort, this optimism was focused on administrative tasks and MSK imaging, reflecting a pragmatic view of AI as a tool to improve efficiency, rather than replace clinical reasoning.

Optimism however co-exists with clear concerns surrounding the use of AI, particularly data security and medical liability, both of which were ranked as high priority by 70% in our survey. These findings are consistent with surveys from Australia, New Zealand and India reporting similar concerns regarding data security and loss of professional expertise [21, 22].

Our thematic analysis identified several critical dimensions regarding AI in rheumatology, specifically concerning safety, trust, governance and implementation challenges (Table 2, Supplementary Data S1). Sentiments fluctuated from cautious optimism to profound apprehension, with some expressing a desire to ‘avoid AI at all costs’ or feeling ‘overwhelmed’ by AI. To date, clinical discourse has been defined by a considerable dichotomy, ranging between overconfidence and intense polarization [23]. These perspectives may be attributed to three primary drivers [23]: the ambiguity and breadth of the ‘AI promise’, which allows for personal assumptions about the threats and opportunities, the ubiquity of AI in popular media that can foster unsubstantiated impressions in broader contexts including its clinical capability, and a systemic desperation for a ‘panacea’ to the health crisis and workforce challenges. As AI continues to evolve in the coming years, responsible integration into rheumatology must involve interdisciplinary collaborative efforts, robust evaluation and ethical oversight to ensure patient safety and trust amongst healthcare professionals.

### Strengths and limitations

The strengths of this work include this being a timely national survey with opinions captured from a broad range of professional roles, therefore providing a comprehensive, multidisciplinary perspective on AI readiness within the specialty. The survey was developed with input from a diverse range of healthcare professionals, ensuring the questions were written in an accessible language and addressed the practical concerns of the UK rheumatology workforce. Consequently, these data provide a timely and essential baseline of digital literacy and AI expectations to inform national education strategies and safe clinical adoption. Although conducted in the UK, the opportunities and challenges identified offer transferable lessons for international institutions navigating similar AI integration hurdles in governance, digital literacy and clinical trust.

Certain limitations must be acknowledged, including a relatively modest sample size which may not be fully representative of the entire UK rheumatology community. As with all optional surveys, the results may be susceptible to selection bias, where individuals with a pre-existing interest in digital technology or AI may have been more inclined to participate.

In addition, item-level attrition occurred, reducing response numbers for later questions. To mitigate non-response bias, partial responses were retained for all sections, with clear denominators presented in the results. While entirely excluding incomplete entries can introduce selection bias, we acknowledge that retaining partial responses carries a converse risk. Individuals who completed the later, AI-specific questions may differ systematically (e.g. possessing greater digital literacy or AI optimism). Consistent patterns across surveys of healthcare workers, mirrored in our own, include optimism and perceived usefulness of AI [21, 24]. This AI optimism especially relates to the use-cases of administrative burden, assisting with documentation and imaging support. These perceived benefits and concerns sit against a backdrop of low–moderate AI literacy and limited clinical usage [21, 24].

### Conclusion

In conclusion, AI is already influencing rheumatology clinical and research practice and is expected to have a growing impact in the coming years. Although clinicians describe high confidence in digital technology, there remain key barriers to the use of AI. Addressing these through targeted education, clear guidance and thoughtful integration into clinical pathways will be essential to the safe and effective adoption of AI in routine rheumatology care. Overall, the findings suggest cautious optimism toward AI in rheumatology, alongside a clear call for improved governance, oversight, clinical guidance and education to support its safe implementation into practice.

## Supplementary Material

keag454_Supplementary_Data

## Data Availability

The data underlying this article will be shared on reasonable request to the corresponding author.
