# Supplementary material for: Perceptions, knowledge and adoption of artificial intelligence in rheumatology: results from a British Society for Rheumatology survey
Source: Rheumatology (Oxford). 2026 Aug 20;65(9):keag454. doi: 10.1093/rheumatology/keag454 (PMC13584210; doi:10.1093/rheumatology/keag454)
Supplement: Supplementary file 1 [file keag454_supplementary_data.docx]

**Supplementary Data S1: Free text comment analysis**

**Current applications of AI in clinical practice**

Many described using AI to improve the accessibility of patient-facing materials, including “*re-wording new patient information leaflets to make them more understandable and patient friendly,”* “*checking clinic letters are appropriate for educational level for the patient,”* and generating *“patient friendly literature”* about a condition or test. Others used AI to *“summarise guidance from approved sources”* and assist with *“reviewing pathophysiology and mechanisms related to sign and symptoms,”* highlighting its current use in supporting clinical learning and rapid information synthesis. Participants also reported using AI to “*assist in written communication*,” generate *“infographics for presentations,”* organise *“large projects into themes,”* and support *“service improvement ideas and developments,”* suggesting that AI is being explored across both clinical and non-clinical professional activities.

**Key Areas for Further Education in AI**

Free text comments expressed interest in further education across a broad range of AI applications, with some respondents requesting training in several areas of AI. These included a better understanding of the use of AI in research, particularly “*hastening retrospective studies”* and broader “*application of AI in research.”* Several respondents highlighted interest in clinically integrated and specialty-specific AI applications, including *“machine learning in image interpretation”*, *“AI-personalised health information,”* better understanding of NHS-approved AI applications, wearable technologies, and systems to reduce time spent on routine tasks such as blood test checking. Respondents also requested the need for education on “*safe, scalable, and sustainable integration into real-world clinical care*,” alongside the need for clinician oversight of AI-generated recommendations.

**Thematic analysis of free text comments**

Many respondents acknowledged the inevitability of AI adoption in healthcare, although one respondent noted, *“AI is a fantastic tool and should be embraced but with caution.”* Other respondents commented on the importance of *“thorough testing”* and *“checking source data.”* There were fewer responses suggesting dissent such as *“avoiding AI at all costs”* and being *“overwhelmed”* by AI.

Safety, accuracy and trust were prominent concerns, as respondents highlighted risks related to hallucinations and the impact on patient safety. Closely related were concerns regarding governance, validation and regulation. Respondents emphasised the need for approved systems before implementation within healthcare settings. As one respondent noted, it would be important for healthcare workers to use *“approved NHS AI tools.”* Respondents also described several potential benefits of AI, particularly in reducing administrative workload and supporting clinical decision-making. For example, one respondent suggested AI could *“help with admin work… free us up for diagnostics.”*

Despite these perceived benefits, respondents identified several implementation challenges, particularly related to funding and workforce readiness. Barriers included integration with existing systems and limited digital literacy within healthcare settings. Many respondents also reflected on the impact of AI on patients. While AI was largely viewed as a tool to support clinicians, some noted the growing influence of AI-generated information on patient expectations. As one respondent observed patients saying *“‘ChatGPT told me…”* Finally, respondents highlighted the importance of future clinician involvement in AI adoption, reiterating that healthcare professionals should help shape how these technologies are implemented.
